# The Positive Role and Mechanism of Herbal Medicine in Parkinson's Disease

**DOI:** 10.1155/2021/9923331

**Published:** 2021-09-03

**Authors:** Rong Yin, Jie Xue, Yanfeng Tan, Chuantao Fang, Chunchun Hu, Qian Yang, Xinyu Mei, Dashi Qi

**Affiliations:** ^1^Institute of Pediatrics, Department of Neonatology, Developmental and Behavioral Pediatric Department & Child Health Care Department, Children's Hospital of Fudan University, Fudan University, Shanghai, China; ^2^Center for Clinical Research and Translational Medicine, Department of Neurology, Yangpu Hospital, Tongji University School of Medicine, Shanghai, China; ^3^Tenth People's Hospital of Tongji University, Tongji University School of Medicine, Shanghai, China

## Abstract

Parkinson's disease (PD) is a complex neurodegenerative disease, manifested by the progressive functional impairment of the midbrain nigral dopaminergic neurons. Due to the unclear underlying pathogenesis, disease-modifying drugs for PD remain elusive. In Asia, such as in China and India, herbal medicines have been used in the treatment of neurodegenerative disease for thousands of years, which recently attracted considerable attention because of the development of curative drugs for PD. In this review, we first summarized the pathogenic factors of PD including protein aggregation, mitochondrial dysfunction, ion accumulation, neuroinflammation, and oxidative stress, and the related recent advances. Secondly, we summarized 32 Chinese herbal medicines (belonging to 24 genera, such as *Acanthopanax*, *Alpinia*, and *Astragalus*), 22 Chinese traditional herbal formulations, and 3 Indian herbal medicines, of which the ethanol/water extraction or main bioactive compounds have been extensively investigated on PD models both *in vitro* and *in vivo*. We elaborately provided pictures of the representative herbs and the structural formula of the bioactive components (such as leutheroside B and astragaloside IV) of the herbal medicines. Also, we specified the potential targets of the bioactive compounds or extractions of herbs in view of the signaling pathways such as PI3K, NF-*κ*B, and AMPK which are implicated in oxidative and inflammatory stress in neurons. We consider that this knowledge of herbal medicines or their bioactive components can be favorable for the development of disease-modifying drugs for PD.

## 1. Introduction

Parkinson's disease (PD), a long-term neurodegenerative disorder of the central nervous system (CNS) that mainly affects the motor system, was first described in “Essay on the Shaking Palsy” by James Parkinson in 1817 [1, 2]. In epidemiology, PD incidences are estimated to range between 5 and 346/100,000 person-years in European countries, which also increases by 5- to 10-fold in populations from 60 to 90 years old [2, 3]. Patients with PD commonly manifest clinical symptoms including tremor, rigidity, slowness of movement, difficulty in walking, autonomic dysfunction, pain, and cognitive decline in the later stages [4–6]. In pathology, the brain tissues of PD patients mostly display the loss of dopaminergic neurons in the substantia nigra pars compacta (SNpc) of the midbrain, the deposit of intraneuronal protein (called Lewy bodies), and aggregates of cytoplasmic inclusions containing insoluble *α*-synuclein [2]. Over the past decades, it has been well documented that oxidative stress, impaired mitochondrial function, inflammation, apoptosis, dysfunction of proteolysis, and loss of neurotrophic factors are implicated in the pathogenesis of PD [7]. In treatment, dopamine replacement and levodopa, two prevalent medications for PD, only exhibit some effects of limited symptomatic relief but cause many severe adverse effects, such as hallucination and involuntary movement [8, 9]. Therefore, disease-modified therapy for PD is currently unavailable.

Herbal medicines, as the fundamental part of traditional medicine (such as in China and India), have been gradually accepted for use in the treatment of various diseases worldwide due to their multilevel function characteristics and remarkable efficacy (in some cases) with fewer adverse effects [10]. For example, natural products derived from Chinese herbal medicines, such as curcumin, epigallocatechin gallate, ginsenosides, berberine, artemisinins, emodin, ursolic acid, silibinin, triptolide, cucurbitacins, oridonin, tanshinone, artesunate, shikonin, *β*-elemene, gambogic acid, cepharanthine, and wogonin, have been demonstrated with multiple bioactivities including proapoptotic, antiangiogenic, and antifibrotic effects, as well as immunity balance, autophagy regulation, and chemotherapy improvement both *in vitro* and *in vivo* [11, 12]. In ancient China, many herbal medicines listed in *Shennong's Classic of Materia Medica*, the earliest complete pharmacopeia of China, are still being practiced in the treatment of PD, such as *Radix achyranthis bidentatae*, *Herba asari*, *Fructus viticis*, and *Fructus xanthii* [13]. In India, there has also been a long history of using herbal medicines in the treatment of neurodegenerative diseases, such as *Withania somnifera*, *Mucuna pruriens*, and *Tinospora cordifolia*. These lines of evidence indicated that herbal medicines may be promising candidates to obtain disease-modifying drugs for PD. In modern pharmacological research, the ingredients or extracts of herbal medicines (such as *Acanthopanax*, *Alpinia*, and *Astragalus*) indeed have been demonstrated to exhibit continuous and considerable effects on the models of PD [14, 15]. Over the past decades, the potential molecular targets of herbal medicine extracts have been extensively discovered, which will facilitate the identification of the bioactive compounds of the pharmacodynamic mechanisms of these herbs [15]. In this review, we will summarize the recent updates in studies that (1) elevate the effects of herbal medicine extracts on PD models and (2) explore the potential working mechanisms or targets of herb extracts or bioactive ingredients. We also included the usage of some common Chinese herbal formulations with considerable anti-Parkinsonian activities. We hope the knowledge may facilitate the development of disease-modifying drugs for PD.

## 2. Pathogenesis of PD

### 2.1. Protein Misfolding and Aggregation

**Figure 1:**
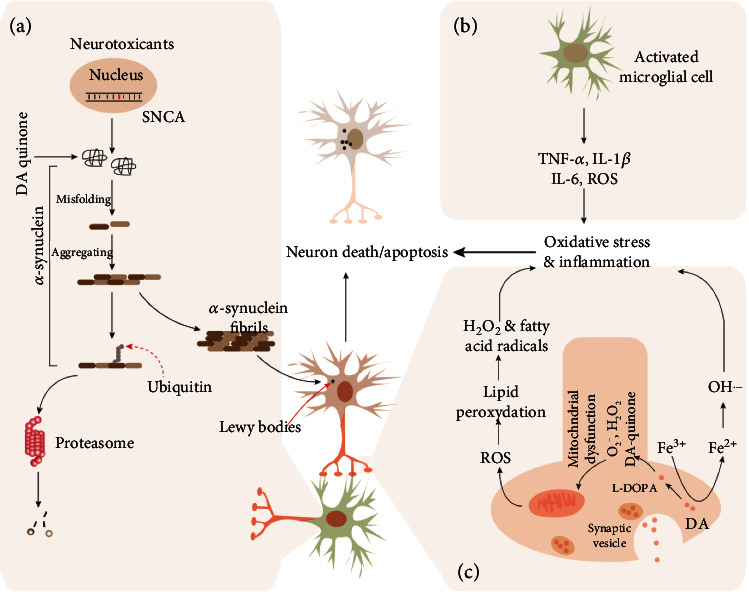
Major mechanisms involved in Parkinson's disease. In the dopaminergic neurons in the substantia nigra pars compacta (SNpc) of the midbrain of patients with Parkinson's disease, mutations in SNCA (coding gene of *α*-synuclein) or protein modification of *α*-synuclein induced by neurotoxicants (or reactive oxygen species) (a) leads to the *α*-synuclein misfolding. The misfolded *α*-synuclein can further aggregate into *α*-synuclein fibrils when the proteasome-mediated degradation system cannot fully clear the fibrils, and then contribute to the production of Lewy bodies in neurons. The inflammatory cytokines, such as TNF-*α*, IL-1*β*, and IL-6, secreted by activated microglial cells (b) also induce the death or apoptosis of neurons. Besides, the mitochondrial dysfunction induced by L-DOPA or Fe^3+^ induces the product of ROS, which enhances death or apoptosis via causing oxidative stress (c). L-DOPA: L-levodopa; ROS: reactive oxygen species.

Although the underlying mechanism remains elusive, protein misfolding and aggregation are the most common molecular phenomena and causative factors for the pathogenesis of PD. For example, the protein of SNCA, PARK2, PINK1, DJ-1, and LRRK2 frequently misfold in the SNpc of the midbrain due to the mutations in their gene [16–18]. Lewy bodies (LBs), a kind of neuronal inclusion, are the aggregation of abnormal proteins in the nerve cells of certain brain regions, which also serve as the major pathological hallmark of PD and dementia [19]. Although *α*-synuclein is the main component of LBs, it also has been found to play critical roles in other Lewy pathologies, such as pale bodies and Lewy neurites [20, 21]. In physiological conditions, *α*-synuclein is naturally present as an unfolded and structured protein, unlikely to transform into highly organized fibrils (Figure 1). However, in the presence of extreme stimuli such as acidic pH and high temperature, it exhibits a strong proneness to transform into a partially folded conformation or intermediate, which intensely promotes the formation of *α*-synuclein fibrils [22–26]. Therefore, a model for the fibrillation of *α*-synuclein was proposed, in which the first step is the conformational transformation of the natively unfolded protein into the aggregation-competent partially folded intermediate.

Consistently, Uversky et al. observed several different aggregated *α*-synuclein forms such as ring-like protofibrillar, amorphous, oligomeric intermediates, amyloid fibrils, and spherical-shaped [27]. In support of the environment-induced pathogenesis of PD, many exogenous chemical compounds such as pesticides, herbicides, and metal ions were demonstrated to accelerate the aggregation process of *α*-synuclein [28, 29]. In another line, multiple missense point mutations (such as A30P, G51D, E46K, A53T, and A30P) of the *α*-synuclein coding gene have been identified in the familial PD cases from different populations including Spanish, Italian-American, and German [22–26], which aggravate the misfolding and aggregation of this protein in the SNpc of patients. Also, the increased accumulation of *α*-synuclein protein was frequently observed in family members of PD patients, suggesting point mutations of *α*-synuclein may be critical risks of its aggregation.

Fujiwara et al. identified a posttranslational modification p-Ser129 of *α*-synuclein, and also found that Ser129 of *α*-synuclein is extensively phosphorylated in synucleinopathy lesions [30]. *In vitro* data by Fujiwara et al. showed that p-Ser129 of *α*-synuclein promotes *α*-synuclein fibril formation [30]. In 2019, Hu et al. found that adenosine triphosphate- (ATP-) dependent Clp protease (ClpP), a mitochondrial matrix protease, suppresses the phosphorylation of *α*-synuclein Ser129 to promote neuronal morphology of neurons derived from PD patients carrying the *α*-synuclein A53T mutant [10]. This finding suggests that ClpP might be a useful therapeutic target for *α*-synuclein-induced neuronal pathologies, such as PD and other synucleinopathies.

Although age is considered the greatest risk factor for *α*-synuclein formation, the underlying details are still exclusive. Based on the evidence that misfolded *α*-synuclein protein is found in both the brain and periphery system of PD patients, Braak et al. have carried out animal experiments to prove that the initial misfolded *α*-synuclein may be formed from nonnerve tissues and then spread to the brain via peripheral autonomic nerves [31]. They found a robust age-dependent gut-to-brain and brain-to-gut spread of *α*-synuclein pathology along the sympathetic and parasympathetic nerves of rats, and *α*-synuclein pathology is more densely packed and resistant to enzymatic digestion in old rats. Their observations indicate that age is a crucial factor for *α*-synuclein aggregation.

### 2.2. Mitochondrial Dysfunction

Mitochondria are the most critical energy-producing center by generating ATP in almost all eukaryotic cells [32]. Over the past several decades, mitochondrial dysfunction (particularly oxidative stress) has been demonstrated to contribute to the pathogenesis of PD by multiple lines of evidence both in PD patients and related animal models [33–35] (Figure 1). MPTP, a synthetic opioid drug produced during the manufacture of 1-methyl-4-phenyl-4-propionoxypiperidine (MPPP), interferes with the components of the mitochondria electron transport chain (ETC) to be transformed into a toxic cation named 1-methyl-4-phenylpyridinium (MPP^+^) via a monoamine oxidase B enzymatic action [36]. In neurons, MPP^+^ efficiently induces oxidative stress (e.g., nitric oxide) and ATP production restrains, which further leads to an elevation of intracellular calcium concentration and excitotoxicity-mediated neuronal damage [37]. Importantly, it was frequently observed that MPTP intake results in mitochondrial dysfunction, and causes permanent PD symptoms among different experimental models [38–40]. In the substantia nigra region of PD patients, the elevation of MPTP metabolites also was frequently observed, which causes the inactivation of ETC components (i.e., complex I) [41–43]. On the other hand, the aberrations of mitochondrial functions, such as rotenone-induced functional inhibition of complex I (rotenone, lipophilic pesticides) also cause PD-related anatomical, behavioral, neurochemical, and neuropathological abnormalities in human patients [44]. Moreover, in patients from familial PD, the maternally inherited mutations in mitochondrial DNA (encoding proteins for the synthesis of ETC components) or 12S rRNA (influencing cytochrome c oxidase production) that lead to mitochondrial dysfunction are tightly associated with the pathogenesis of PD [45, 46].

Recently, many researchers tried to explain the pathogenesis of PD in the view of mitochondria-lysosome crosstalk. In 2021, Kim et al. observed that mitochondria-lysosome contacts were dynamic in the soma, axons, and dendrites of human neurons [47]. Whereas, it exhibited a morphological contact prolongation in the neurons derived from PD patients that harbor mutant GBA1 [47]. They also demonstrated that the prolongation was due to the decreased GBA1 lysosomal enzyme activity because the phenotype could be rescued by restoring enzyme activity with a GCase modulator. Furthermore, the contact prolongation resulted in the disruption of mitochondrial distribution and function. Therefore, all the observations definitely indicate the association between mitochondrial dysfunction and PD. More recently, a study by Matsui et al. showed that cytosolic double-strand DNA (dsDNA) of mitochondrial origin escaping from lysosomal degradation exhibits cytotoxicity in cultured cells and PD phenotypes *in vivo* [48]. The cytotoxicity was largely neutralized by the overexpression of DNase II (a lysosomal DNase that degrades discarded mitochondrial DNA) or the depletion of IFI16 (a sensor for cytosolic dsDNA of mitochondrial origin). Moreover, reducing cytosolic dsDNA by overexpressing human DNase II ameliorates movement disorders and dopaminergic cell loss in GBA-mutated PD zebrafish models. These results support a common causative role for the cytosolic leakage of mitochondrial DNA in PD pathogenesis.

### 2.3. Unbalance of Metal Ion Homeostasis in the Brain

In physiological conditions, ions (in particular calcium and iron) have been explicitly demonstrated to be implicated in various vital biological processes including DNA biosynthesis, myelin sheath and neurotransmitters, mitochondrial respiration, and brain development and metabolism [49–51]. The accumulation of iron in the SNpc and reticulata of PD patients has been frequently observed, which also increases with disease severity [52–56] (Figure 1). In 2017, Lei et al. found that, in mice, lithium administration induces the elevation of nigral and cortical iron by lowering brain tau levels, thereby leading animals to show cognitive loss and parkinsonian features [57]. Besides, single nucleotide polymorphisms or mutations in DMT1 (divalent metal transporter 1, involving iron transportation) were identified in dopaminergic neurons of PD patients [58–60]. In 2020, Angelova et al. reported that ferroptosis, an iron-dependent form of necrotic cell death marked by oxidative damage to phospholipids, participates in the pathogenesis of PD in human iPSC-derived neurons [61]. Generally, ferroptosis causes the accumulation of 15-hydroperoxy Hp-arachidonoyl phosphatidylethanolamine (15-HpETE-PE) which can induce a death signal. In fibroblasts from a patient with a PD-associated mutation (fPDR747W), Sun et al. recently found a selective elevation in 15-HpETE-PE level sensitivity to ferroptosis [62]. They also constructed Pnpla9R748W/R748W (mutations relate to neurodegeneration in human) mice using CRISPR/Cas9 technology and observed that the mice exhibited progressive parkinsonian motor deficits and 15-HpETE-PE accumulation. Meanwhile, they provided evidence to support that 15-HpETE-PE level is elevated in midbrains of rotenone-treated PD rats and *α*-synuclein-mutant A53T mice. These observations indicate that iron ion homeostasis is required for the physiological functions of the brain.

In another line, the cytosolic Ca^2+^ in SNpc DA neurons is mainly responsible for three complementary functions: (1) helps maintain the slow tonic spiking in these neurons, even though it is not required for pacemaking; (2) positively modulates the expression and activity of enzymes involved in DA synthesis, ensuring a match between the supply and demand of the neurotransmitter; and (3) stimulates oxidative phosphorylation and ATP production [63–65]. CaV1.3, a subtype of Ca^2+^ channel, was found to be used in dopaminergic neurons vulnerable to neurodegeneration in the SNpc of adult (but not juvenile) mice for the pacemaking activity of the neurons [64, 66]. Several studies by independent groups indicated that, in SNpc dopaminergic neurons of PD patients with mitochondrial dysfunction, CaV1.3 channels make cells more susceptible to Ca^2+^-mediated excitotoxicity [66, 67]. Besides, benidipine, an FDA-approved drug that functions as a voltage-gated calcium channel antagonist, was recently identified to suppress rotenone-induced apoptosis in DA neurons. These studies indicate that the dysregulation of calcium homeostasis may be a critical factor for PD pathogenesis.

### 2.4. Inhibition of Proteasome-Mediated Degradation

The proteasome is an extremely vital molecular apparatus that ubiquitously locates in the nucleus and cytoplasm of eukaryotic cells, which degrades unwanted or misfolding proteins with ploy-ubiquitin modifications via its protease activity [68]. It is well accepted that an abnormal ubiquitin-proteasome system (UPS) is tightly associated with PD symptoms [69, 70] (Figure 1). Previously, the upstreams of UPS, BDNF (brain-derived neurotrophic factor), and its receptor TRKB (tyrosine kinase B) were demonstrated to regulate the expression of key synaptic proteins in response to neuronal activity, which is also considered to play vital roles in the pathogenesis of PD [71]. PARK2, a gene coding the essential ubiquitin ligase enzyme of UPS, has been found with several types of mutations including missense, frameshift, nonsense, point mutations, exon deletions, and duplications in PD patients [7, 71, 72]. PARK7, encoding a protein that inhibits *α*-synuclein aggregation, also was reported that its mutations increase the susceptibility to proteasome inhibition and enhance oxidative stress in neurons [73]. FBXO7 is a clinically relevant F-box protein linked to early-onset PD, in which mutations near the F-box domain and substrate recruiting domains were reported to influence SCF^FBXO7^/PARK15 ubiquitin ligase activity. In 2016, Teixeira et al. conducted a high-throughput screen to identify the ubiquitinated substrates of SCF^FBXO7^ that may be directly involved in PD etiology [74]. They validated GSK3*β* (glycogen synthase kinase 3*β*, a kinase of *α*-synuclein) and TOMM20 (translocase of outer mitochondrial membrane 20, a mitochondrial translocase) as SCF^FBXO7^ substrates both *in vitro* and *in vivo*. Although it promoted K63 ubiquitination of GSK3*β*, it was found that FBXO7 failed to affect the protein level and localization of endogenous GSK3*β*. Besides, they reported that ectopic FBXO7 with mutants associated with early-onset PD could not alter the ubiquitination level of TOMM2. Therefore, whether GSK3*Β*/TOMM2 involves the pathological processes of PD remains ambiguous.

### 2.5. Neuroinflammation

Both innate and adaptive immune responses have been demonstrated to involve the pathophysiology of PD [75] (Figure 1). For example, the expression level of nuclearly translocated NF-*κ*B (nuclear factor kappa-light-chain enhancer of activated B cells) was reported to be increased in the dopaminergic neurons of PD patients [76]. In the cerebrospinal fluid and striatum of PD patients, the increment of cytokine levels, such as T-cell activation-associated cytokine (IL-2), proinflammatory cytokines (TNF-*α*, IL-1*β*, and IL-6), anti-inflammatory cytokine (IL-4), and several growth factors (EGF and TGF-*β*1), is the main feature of inflammation-induced processes [77, 78]. In MPTP-induced PD rats, mice, and monkeys, the increased astroglial reaction and microglial activation also were observed in both the SNpc and the striatum [79–81]. Recently, an *in vivo* study in Tlr4-knockout mice by Perez et al. showed that Tlr4-mediated inflammation plays an important role in intestinal and/or brain inflammation, which may be one of the key factors leading to neurodegeneration in PD [82]. Overall, these findings support the hypothesis that inflammatory cytokines are produced in the dopaminergic neurons that play potentially vital roles in the pathogenesis of PD.

On the other hand, Brochard et al. found the increased amounts of CD8^+^ T-cytotoxic and CD4^+^ T-helper cell infiltration in the nigrostriatal system of MPTP-injected mice [83]. In MPTP-exposed PD patients, there is an elevated expression of Fas ligand, a cell-surface ligand of the TNF-*α* family that triggers the Fas receptor and induces apoptosis, within the striatum and SNpc [84]. Another neuroinflammatory modification in PD is the increased expression of major histocompatibility complex (MHC), the molecules that bind to the pathogen-derived peptide fragments exposed on the cell surface [85]. Initially, McGeer et al. observed that the number of HLA-DR-positive microglial cells (MHC-II) is significantly increased in the SNpc of PD patients [86]. Similarly, the increased level of light chain MHC-I also was observed in the striatum of PD patients compared with normal controls [87]. Besides, Bokor et al. found that killer cells induced by antibody-dependent cell-mediated cytotoxicity reaction also play roles in the pathogenesis of PD [88]. Recently, Sulzer et al. showed that a defined set of peptides that are derived from *α*-synuclein act as antigenic epitopes displayed by these alleles and drive helper and cytotoxic T-cell responses in PD patients [89]. Previously, circulating CD4^+^ and CD8^+^ T-cells derived from PD patients have been demonstrated to produce Th1/Th2 cytokines in the presence of *α*-synuclein, suggesting that chronic memory T cell response may exist in PD. In 2021, Williams et al. generated an *α*-synuclein overexpression and T cell-deficient mouse model to elucidate whether *α*-synuclein aggregation in the midbrain of mice can induce memory T cells to lead to PD [90]. Indeed, they observed that *α*-synuclein overexpression upregulates the MHC-II protein level in CNS myeloid cells and induces infiltration of IFN*γ*-producing CD4^+^ and CD8^+^ T cells into the CNS. More importantly, loss of function of TCR*β* or CD4 using the immunosuppressive drug fingolimod could reduce the CNS myeloid MHC-II response to *α*-synuclein. All the observations highlight the critical roles of inflammation in the pathogenesis of PD.

### 2.6. Oxidative Stress

In human bodies, oxidative stress occurs when the production of reactive oxygen species (ROS) cannot be neutralized by antioxidants, and often leads to the damage of cellular components including lipids, proteins, and DNA. Numerous experimental studies in dopamine metabolism, lipid peroxidation (LPO), and glutathione depletion have demonstrated that oxidative stress plays a critical role in the pathogenesis of PD (Figure 1). In dopaminergic neurons of the SNpc, DA metabolism generates various oxidative byproducts including O^−^ (superoxide anion), H_2_O_2_ (hydrogen peroxide), and DA quinone species, which can modify cellular nucleophiles including low molecular weight sulfhydryls (e.g., GSH) and protein cysteinyl residues [91]. It has been demonstrated that DA quinones are implicated in PD pathophysiology by modifying proteins including *α*-synuclein, parkin, SOD2, DJ-1, and UCH-L1 [92–94]. Also, DA quinone species cause the dysfunction of brain mitochondrial respiration and lead to ROS production by altering the subunits of the ETC (complexes I and III) [95, 96].

Lipid peroxide (LPO) in the plasma membrane is capable of removing hydrogen atoms from the methylene bridges (-C_2_H_2_-) to produce H_2_O_2_ and fatty acid radicals. In the substantia nigra of patients with Parkinson's disease, the level of basal malondialdehyde, an intermediate in the production of LPO, was previously reported to be increased significantly when compared with other brain regions, suggesting that LPO may participate in the development of PD [95, 97]. Also, the end products of LPO, such as 4-hydroxynonenal and thiobarbituric acid reactive substance, are increased in the substantia nigra and striatum of PD brains [98, 99]. Recently, Jiang et al. reported that *Tianma-Gouteng granules* significantly decrease the susceptibility of PD by inhibiting ALOX15-mediated lipid peroxidation, suggesting that intervention by targeting LPO production may be an effective therapy for PD [100]. Oxidative stress was previously reported to activate the integrated stress response, which further ignites activating ATF4 (transcription factor 4). In 2021, Demmings et al. explored the role of ATF4 in neuronal cell death in MPP^+^- and (6-hydroxydopamine-) 6-OHDA-induced PD mouse models and found that *α*-synuclein aggregation could cause significant elevation of ATF4 expression in mouse cortical and mesencephalic dopaminergic neurons [101]. Furthermore, they demonstrated that neuronal death induced by PD neurotoxin and *α*-synuclein fibrils is attenuated in ATF4-deficient dopaminergic neurons, and ectopic expression of ATF4 restores sensitivity of ATF4-deficient neurons to PD neurotoxins. These results collectively indicate the key roles of oxidative stress in the pathogenesis of PD.

Glutathione (GSH), a critical “scavenger” of ROS such as free radicals, peroxides, and LPO in cells, is expressed at a relatively low level in the substantia nigra when compared with other brain regions such as the cortex, hippocampus, and cerebellum [102]. In early 1992, Sofic et al. reported that, compared with the control subjects, the level of GSH in the substantia nigra of PD patients is significantly decreased [103]. Nandita et al. demonstrated that the early GSH losses in the substantia nigra may directly cause a reduction in the activity of ETC complex I, which results in dopaminergic cell death and eventually promotes the development of PD [104]. Furthermore, the depletion of GSH also causes the dysfunction of the UPS, and thereby deprives the 26S proteasome protein degradation system in neurons of PD [104]. Besides, GSH depletion induced inflammation stress in neuronal tissues of PD patients by modulating IL-1 signaling and JNK- (c-Jun N-terminal kinase-) activated inflammatory pathways [105, 106].

## 3. Chinese Herbal Medicines and PD

### 3.1. Acanthopanax

**Figure 2:**
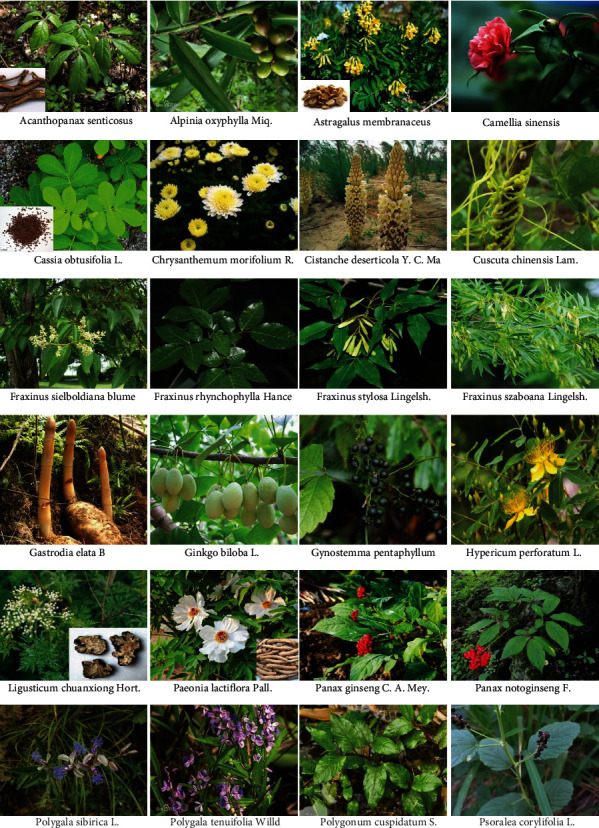
Representative of Chinese herbal medicine for Parkinson's disease

**Figure 3:**
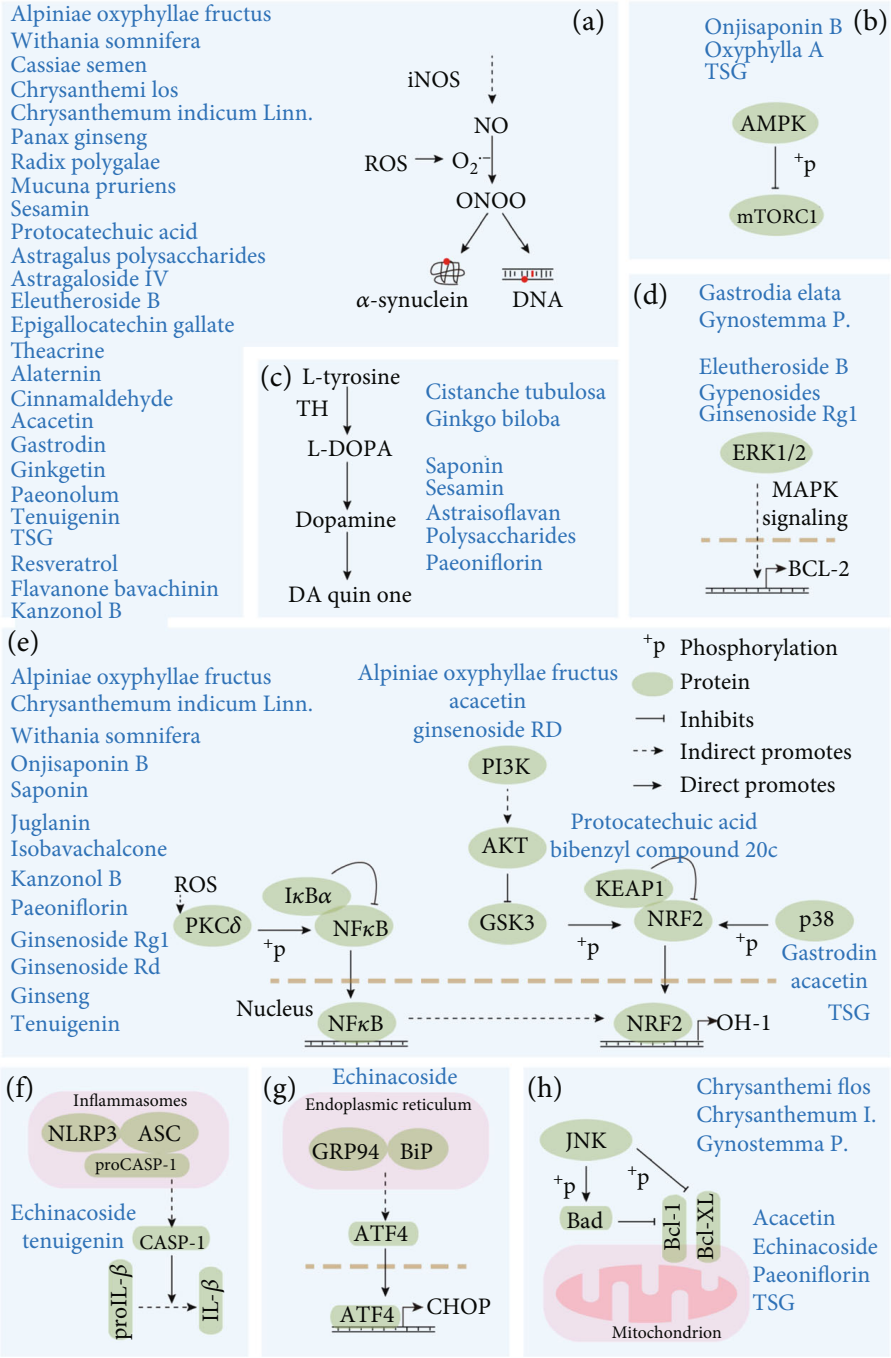
Main signaling pathways involved in Parkinson's disease that are targeted by herbs or their bioactive ingredients. (a) The generation of ROS and their toxicity to neurons. iNOS: inducible nitric oxide synthase. (b) AMPK/mTOR signaling pathway. (c) The metabolism of DOPA. (d) ERK/MAPK signaling pathway. (e) The crosstalk of the NF-*κ*B, PI3K, NRF2, and p38 MAPK pathways. (f) CASP-1/IL-1*β* signaling pathway. (g) Grp94/Bip/ATF4 signaling pathway. (h) JNK-Bcl2/XL singaling pathway.

**Figure 4:**
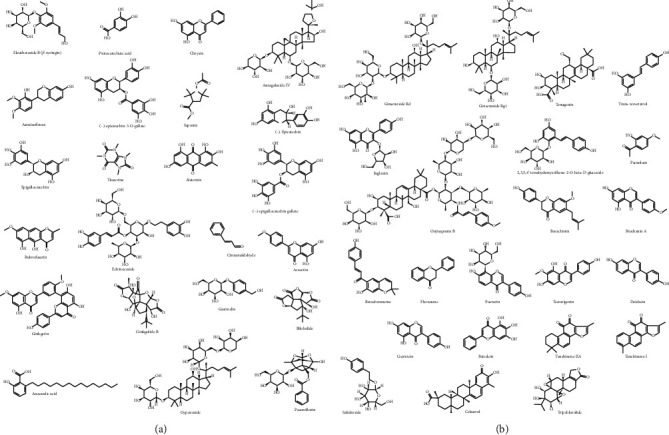
(a) Chemical structural formula of the main bioactivity components derived from Chinese herbal medicine for Parkinson's disease. (b) Chemical structural formula of the main bioactivity components derived from Chinese herbal medicine for Parkinson's disease.

*Acanthopanax senticosus* roots and stems (ASRS), also named Wujipi in Chinese, are widely used in traditional Chinese medicine. The pole-climbing test showed that the ethanol extracts (45.5 mg/kg daily) of *Acanthopanax senticosus* (Figure 2) roots possess neuroprotective effects on MPTP-induced PD mice [107]. In pathology, the number of dopamine receptor D1/2-positive cells and caspase-3 protein levels of substantia nigra were significantly reduced after the administration of the extract. Sesamin, a component of *Acanthopanax senticosus* roots, pharmacologically offers protective effects against PD-related depressive behaviors in rotenone-administered rats by enhancing tyrosine hydroxylase or glial cell line-derived neurotrophic factor- (GDNF-) positive neuron activity in the midbrain [108, 109]. Lahaie et al. observed that sesamin also elicits a strong elevation of SOD activity and decreases catalase activity and synthase protein level of nitric oxide (NO) in MPP^+^-induced neuronal PC12 cells [110] (Figure 3). Eleutheroside B (Figure 4), another main component of ASRS, can also relieve fatigue, enhance memory, and improve human cognition. In MPP^+^-induced PC12 cells, eleutheroside B effectively increases the phosphorylation of ERK1/2 (extracellular signal-regulated kinase 1/2) and reduces the expression level of c-Fos and c-Jun [111] (Figure 3). In 2016, Li et al. carried out lncRNA microarray analysis to systematically investigate the effects of ASRS on the CNS both in pathology and physiology [112]. However, they observed that ASRS fails to inhibit *α*-synucleinopathies but produces some potential neurotoxicity to CNS under physiological conditions, indicated by no significant difference in the expression of lncRNA/mRNA that may cause potential neurotoxicity analogous to *α*-synuclein that exists between ASRS-treated and -untreated *α*-synuclein mice in physiological conditions [113]. These findings hint that, in different situations, the bioactivities of ASRS may be bidirectional for pathological and physiological CNS.

### 3.2. Alpinia

*Alpiniae Oxyphyllae* Fructus (AOF, known as YizhiRen in Chinese), the dried, ripe seed of *Alpinia oxyphylla* Miq. (Figure 2), is commonly practiced in clinics to strengthen the spleen, stomach, and kidney functions and cure vomiting, diarrhea, cold pain in the abdomen, excessive salivation, etc. [114]. Ethanol extract of AOF was reported to restore 6-OHDA-induced dopaminergic neuron degeneration and attenuate a deficit of locomotor activity in a zebrafish model of PD by alleviating inflammation (downregulation of IL-1*β* and TNF-*α* expression) and oxidation (inhibition of NO production) stress [115] (Figure 3). Moreover, AOF achieves its bioactivities in neuroprotection partially via the PI3K-AKT pathway [115] (Figure 3). In 2015, Zhang et al. identified two polyphenols including protocatechuic acid and chrysin (Figure 4) from AOF, and demonstrated that these two polyphenols synergistically enhance cell viability in 6-OHDA-treated PC12 cells and significantly attenuate dopaminergic neuron loss in both zebrafish and mice PD models [116]. In mechanisms, they proved that protocatechuic acid and chrysin (1) increase NRF2 (nuclear factor-erythroid 2-related factor 2) protein level and transcriptional activity, (2) modulate cellular redox status, and (3) decrease levels of malondialdehyde [116] (Figure 3). Oxyphylla A, a bioactive compound from AOF has promising neuroprotective effects: (1) it ameliorates chemical-induced primary neuron damage *in vitro*, and (2) it alleviates the chemical-induced dopaminergic neuron loss and behavioral impairment *in vivo* [113] (Figure 3). Recent research reported that oxyphylla A significantly promotes *α*-synuclein degradation in a cellular PD model via activating the PKA-AKT-mTOR pathway to trigger PSMB8 expression and enhance UPS activity [117] (Figure 3). Moreover, it also reduces the accumulation of both triton-soluble and -insoluble forms of *α*-synuclein to protect neurons against *α*-synuclein-induced neurotoxicity in A53T *α*-synuclein transgenic mice [117].

### 3.3. Astragalus

*Astragali Radix* (Huangqi in Chinese), the dried root of *Astragalus membranaceus* (Fisch.) Bge. var. mongholicus (Bge.) Hsiao or *Astragalus membranaceus* (Fisch.) Bge. (Leguminosae), is a common and well-known drug in traditional Chinese medicine [118] (Figure 2). Currently, at least three bioactive compounds including astragalus polysaccharides, astraisoflavan, and astragaloside IV (Figure 4) have been identified to possess neuroprotective functions in *Astragali Radix*. Although the reason for the death of neurons in PD patients is unclear, oxidative stress such as free radicals obviously contributes to the development of this disease [119]. Astragalus polysaccharides have been identified to relieve oxidative stress in dopaminergic neurons [120] (Figure 3). In MPP^+^-treated SH-SY5Y cells, astragaloside IV significantly reverses the loss of cell viability, nuclear condensation, the generation of intracellular ROS, and the elevation of Bax/Bcl-2 ratio and caspase-3 activity [121] (Figure 3). Neural stem cells (NSCs) are important cellular sources of transplantation therapies for PD patients. Gao et al. also systematically estimated the protective effects of astraisoflavan and astragalus polysaccharides on NSCs and found that these ingredients significantly promote the expressions of tyrosine hydroxylase and dopamine transporter in dopamine neurons and the motivators of dopamine neurons including Shh (sonic hedgehog), Nurr1 (orphan nuclear hormone 1), and Ptx3 (pituitary homeobox 3) [122].

### 3.4. Camellia

Camellia (also known as Green Tea in Chinese) is the product derived from the leaves of *Camellia sinensis* (L.) O. Kuntze (Theaceae) (Figure 2). Historically, the infusion of camellia was generally used as a relaxant or detoxifying agent to cure stomach problems, headaches, and nervous tension [123]. In modern pharmacology, green tea polyphenols (such epicatechin, epicatechin gallate, epigallocatechin, and epigallocatechin gallate) (Figure 4) have been shown with several health benefits including antioxidant, anti-inflammatory, and neuroprotective activities [124] (Figure 3). In 6-OHDA-induced PD rats, standardized extracts of camellia seeds, epicatechin, and epigallocatechin gallate obviously revert the behavioral injury, alleviate depression, and improve cognitive function of animals [125]. Also, epigallocatechin gallate treatment provides protection and prevention from the neurotoxicant paraquat- (PQ-) induced reduction in the lifespan and locomotor activity and from the PQ-induced increase in lipid peroxidation and neurodegeneration in *Drosophila melanogaster* flies [126]. Saponin (Figure 4), another major active compound of camellia seeds, increases dopamine content in the striatum and tyrosine hydroxylase-positive cells in substantia nigra and relieves inflammation and behavioral disorder in MPTP-induced PD mice [127] (Figure 3). More recently, Duan et al. investigated the protective functions of theacrine (a purine alkaloid from camellia, Figure 4) in multiple animal models of PD, and found it reverts the loss of dopaminergic neurons and the damages of behavioral performance [128]. In the mechanism, they illustrated that theacrine directly activates SIRT3 to promote SOD2 deacetylation, which reduces ROS accumulation and restores mitochondrial function [128] (Figure 3).

### 3.5. Cassia

*Cassiae Semen* (Juemingzi in Chinese) is the dried, ripe seed of *Cassia obtusifolia* L. or *Cassia tora* L. (Leguminosae) (Figure 2). In ancient China, it was used to treat dizziness and headaches and provided a benefit to the eyes by anchoring and nourishing the liver [129]. In 6-OHDA-treated PC12 cells, the total ethanol extracts of *Cassiae Semen* were found to attenuate the overproduction of ROS, glutathione depletion, mitochondrial membrane depolarization, and caspase-3 activation [130] (Figure 3). Moreover, *Cassiae Semen* also significantly protected dopaminergic neuronal degeneration in the substantia nigra and striatum of MPTP-treated mice [130]. Peroxynitrite (ONOO^−^), a critical oxidant with reaction with various cellular constituents including lipids, amino acids, sulfhydryls, and nucleotides, has been reported to contribute to the pathogenesis of PD [131]. Alaternin (Table 1), a phenolic active component of *Cassia tora* L., was reported to function as potent ONOO^−^ scavengers to decrease the ONOO^−^-mediated nitration of tyrosine through electron donation [132] (Figure 3). Cinnamaldehyde (Table 1) (at a dose of 5 and 10 *μ*M for 24 h), another critical bioactive component of *Cassia tora* L., was also found to significantly increase the viability and decrease the ROS content of 6-OHDA-treated PC12 cells [133] (Figure 3).

### 3.6. Chrysanthemum

*Chrysanthemi Flos* (Jvhua in Chinese) is the dried flowering head of *Chrysanthemum morifolium* R (Figure 2). In MPP^+^-treated human SH-SY5Y cells, water extracts of *Chrysanthemi Flos* effectively improve cell viability and attenuate the elevated ROS level, Bax/Bcl-2 ratio, and cleavage of caspase-3 [134] (Figure 3). Similarly, Kim et al. found that water extracts of *Chrysanthemum indicum* Linn. also protect SH-SY5Y cells from MPP^+^-induced damage by retarding ROS production, elevating of the Bcl-2/Bax ratio, and by PARP (poly-ADP-ribose polymerase) proteolysis [135]. Besides, they also observed that the water extracts block I*κ*B-*α* degradation and activation of NF-*κ*B (p65), thereby limiting inflammation in BV2 cells [135] (Figure 3). Acacetin (5,7-dihydroxy-4-methoxyflavone, Table 1), a flavonoid compound of *Chrysanthemum*, has been proved to be effective in preventing 6-OHDA-induced neuronal cell death through inhibiting mitochondrial-mediated cascade apoptotic cell death and ROS production [136] (Figure 3). Through its mechanism, acacetin also markedly reduces phosphorylation of JNK, p38 MAPK (mitogen-activated protein kinase), PI3K (phosphatidylinositol 3-kinase), and GSK3*β* (glycogen synthase kinase 3*β*) [137] (Figure 3).

### 3.7. Cistanche

Echinacoside (Table 1), a phenylethanoid glycoside isolated from the stems of *Cistanche deserticola* (Figure 2), significantly attenuates cell viability, oxidative stress, and mitochondria-mediated apoptosis and maintains mitochondrial membrane potential (MMP) in 6-OHDA-reduced PC12 cells [138] (Figure 3). In MPTP-induced PD mice, *Cistanche tubulosa* nanopowder effectively improves the cell viability, increases tyrosine hydroxylase expression, and reduces the number of apoptotic cells [139]. Through its molecular mechanism, the treatment of *Cistanche tubulosa* nanopowder increases the protein expression of GDNF, GFR*α*1, and Ret in neurons of the substantia nigra of mice [139]. In 2017, Zhang et al. demonstrated that the echinacoside extracted from *Cistanche deserticola* rescues cells from 6-OHDA-induced ER and oxidative stress *in vivo* [140]. Furthermore, they showed that echinacoside attenuates seipinopathy by promoting seipin degradation by influencing the Grp94/Bip-ATF4-CHOP signal pathway [141]. In contrast, Chen et al. evidenced that echinacoside binds to Sirt1 directly and affects FoxO expression to enhance autophagy in neurons [142]. Recently, echinacoside was observed to inhibit the activation of microglia-mediated NLRP3/CASP-1/IL-1*β* inflammatory signaling to promote dopamine neuron survival in the MPTP-induced PD mice [143] (Figure 3). All these findings indicate that echinacoside may be a multiple-target drug for PD.

### 3.8. Gastrodia

*Gastrodia elata* (Figure 2) Blume is one of the most important traditional plants in oriental countries, of which the active constituents include gastrodin and bibenzyl compound 20c (20c) [100, 144–147] (Table 1). Accumulating observations have shown that gastrodin protects dopaminergic neurons via cleaning free radicals and restraining apoptosis both *in vitro* and *in vivo* [100, 145, 148], while the 20c component alleviates the accumulation of *α*-synuclein, ER stress, and intracellular ROS production *in vitro* [144, 146, 149] (Figure 3). As we all know, L-DOPA is the gold-standard drug for PD, but long-term treatment results in L-DOPA-induced dyskinesia. The water extract of *Gastrodia elata* alleviates L-DOPA-induced axial, limb, orolingual, and locomotive dyskinesia compared to the dyskinesia group via blunting the elevation of pERK1/2 and FosB [150] (Figure 3). Similarly, in MPP^+^-induced SH-SY5Y cells, gastrodin can activate the p38 MAPK/NRF2 signaling pathway to induce HO-1 expression and thereby rescue dopaminergic cells [145] (Figure 3). Also, 20c protects PC12 cells from rotenone-induced apoptosis, at least in part, via activation of the NRF2/ARE/HO-1 signaling pathway [144, 149] (Figure 3). Ferroptosis, a form of necrosis caused by the iron-induced accumulation of lipid hydroperoxide and mediated by glutathione peroxidase activity, has been proven to involve several molecular events during PD development. Recently, gastrodin was observed to increase the protein expression of NRF2, GPX4, ferroportin-1, and HO-1 in H_2_O_2_-treated C6 cells [100].

### 3.9. Ginkgo

*Ginkgo biloba* (Figure 2) extract EGb761 improves memory loss and cognitive impairments in patients with senile dementia, and promotes the proliferation of NSCs in the subventricular zone of PD animals [151–154]. Wang et al. found that ginkgetin (Table 1), a natural biflavonoid isolated from leaves of *Ginkgo biloba*, decreases the levels of intracellular ROS and maintains MMP in MPP^+^-induced PD models both *in vitro* and *in vivo* [138]. Also, they demonstrated that ginkgetin dramatically inhibits MPP^+^-induced cell apoptosis via the caspase-3 and Bc-l2/Bax pathway, strongly chelates ferrous ion to downregulate L-ferritin, and upregulates the level of transferrin receptor 1 [155]. Ginkgolide B and bilobalide (Table 1), two critical bioactive ingredients of *Ginkgo biloba*, enhance cell viability and reduce cell apoptosis in SY5Y cells with recombinant monomeric or aggregated *α*-synuclein *in vitro* [156]. Consistently, in A53T *α*-synuclein transgenic PD mice, the treatment of *Ginkgo biloba* extract improves locomotor activity, inhibits the expression of methane dicarboxylic aldehyde, and recovers the expression of tyrosine hydroxylase and dopamine transporters [157]. In rotenone-induced PD mice, the oral supplement of *Ginkgo biloba* extract also reduces the elevated oxidative and inflammatory stress [158]. Gnkgolic acid, a natural compound extracted from *Ginkgo biloba* leaves, was revealed to significantly decrease intracytoplasmic *α*-synuclein aggregates and SUMO-1 level and increase the number of autophagosomes [159]. More recently, Wu et al. found that protocatechuic acid (Table 1), a component of *Ginkgo bilob*a, increases the efficacy of ginkgolide B in the treatment of PD, suggesting a new idea to efficiently utilize the components of *Ginkgo bilob*a leaves in the treatment of PD [160].

### 3.10. Gynostemma

The ethanol extract of *Gynostemma pentaphyllum* (GP-EX, Figure 2) effectively attenuates cell cytotoxicity and apoptosis and improves cell viability both in MPP^+^-induced cellular and MPTP-lesioned PD mouse model [137, 161]. In 2020, Park et al. reestimated the neuroprotective effects of GP-EX on an A53T *α*-synuclein transgenic mouse model of PD (A53T mice), and found that GP-EX obviously reversed the increased *α*-synuclein-immunopositive cells and *α*-synuclein phosphorylation in the midbrain of A53T mice [162]. In pathology, they observed that GP-EX reverses the *α*-synuclein-reduced phosphorylation of tyrosine hydroxylase, ERK1/2, Bad (Bcl-2-associated death promoter, at Ser112), and JNK1/2[162] (Figure 3). Gypenosides (Table 1), a saponin extract derived from *Gynostemma pentaphyllum*, was demonstrated to ameliorate anxiety disorders in the MPTP-lesioned PD mouse model [163, 164]. In L-DOPA-induced PD animals, gypenoside treatment also alleviates the deficits in habit learning and spatial memory, and dyskinesia [149, 165]. Through its mechanism, gypenoside was frequently found to modulate ERK1/2 phosphorylation in hippocampus tissues [149, 165] (Figure 3).

### 3.11. Paeonia

Paeoniflorin (Table 1), a monoterpene glycoside isolated from the aqueous extract of *Paeoniae Alba* Radix (Figure 2), was found to enhance the autophagic degradation of *α*-synuclein by regulating the expression and activity of ASICs (acid-sensing ion channels) and thus produces protective effects against cytotoxicity [166]. Another group also found that paeoniflorin has a neuroprotective effect on glutamate- or MPP^+^-treated PC12 cells via regulating the MMP and Bcl-2/Bax signal pathway [167, 168] (Figure 3). In 6-OHDA-induced PC12 cells, Dong et al. reported that paeoniflorin inhibits cell apoptosis by, at least in part, inhibiting the ROS/PKC*δ*/NF-*κ*B signaling pathway [169] (Figure 3). In the MPTP-treated mouse model of PD, paeoniflorin treatment ameliorates the behavioral deficits and reduces dopaminergic cell loss [170]. Moreover, paeoniflorin promotes dopamine catabolism and turnover, which partially depends on that protein level decrease of dopaminergic transporter and tyrosine hydroxylase in the striatum and substantia nigra of the PD mice which is largely reversed after paeoniflorin treatment [170]. *Moutan Cortex* Radicis, also called *Moutan peony*, is the root cortex of *Paeonia suffruticosa* Andrews. Ethanol extract of *Moutan Cortex* Radicis alleviates PD-like motor symptoms including increased locomotor activity and reduced bradykinesia of MPTP-induced PD mice [171]. Paeonolum (Table 1), the main component of *Moutan cortex* Radicis, also protects MPP^+^-induced PD zebrafish models against DA neurodegeneration and locomotor dysfunction [172]. In PC12 cells, it also attenuates MPP^+^-induced intracellular ROS accumulation, restores the level of total GSH, and inhibits the mitochondrial cell death pathway [172]. More recently, Xue et al. reported that gold nanoparticles using the root extract of *Paeonia mountan* potentially inhibit the inflammation *in vitro* of murine microglial BV2 and improve motor coordination in PD mice [173].

### 3.12. Panax

In MPP^+^-treated SH-SY5Y cells, the water extract of ginseng (*Panax ginseng* C.A. Meyer, Figure 2) exhibits an inhibitory effect on cell death, ROS overproduction, Bax/Bcl-2 ratio elevation, cytochrome c release, and caspase-3 activation [174] (Figure 3). Panaxatriol saponins, the main constituents extracted from *Panax notoginseng* (Figure 2), provide neuroprotection against the loss of dopaminergic neurons and behavioral impairment caused by MPTP treatment *in vivo* [175]. In *β*-sitosterol-*β*-D-glucoside-triggered progressive PD rats, oral administration of *Panax ginseng* extract reduces dopaminergic cell loss, microgliosis, and accumulation of *α*-synuclein aggregates, and fully prevents the development of locomotor deficits [176]. Ginsenoside Rg1 (Table 1), a natural product extracted from *Panax ginseng*, has been reported to exert notable neuroprotective activities by suppressing phosphorylation and nuclear translocation of NF-*κ*B/p65 and activation of AKT and ERK1/2 in H_2_O_2_-treated PC12 cells [177] (Figure 3). Similarly, Liu et al., also found that ginsenoside Rd (Table 1), one of the main active monomer compounds of the *Panax ginseng* plant, reverses the loss of tyrosine hydroxylase-positive cells in substantia nigra of MPTP-treated mice by modulating the PI3K/AKT survival-signaling pathway [178] (Figure 3). In the rotenone-induced SH-SY5Y cells, ginsenosides also upregulate SOD and aconitase enzyme activities, attenuate the extent of depolarization of MMP, and restore calcium levels [179] (Figure 3). Also, Korean red ginseng was reported to have biological effects like the antioxidant and anti-inflammatory activities in different PD animal models by involving multiple mechanisms including the NF-*κβ* inflammatory pathway, caspase-3-mediated apoptosis, and unfolded protein response [34, 180, 181] (Figure 3).

### 3.13. Polygala

The water extract of *Radix Polygalae*, the root of *Polygala tenuifolia* (Figure 2), was demonstrated to significantly inhibit 6-OHDA-induced cell damage, caspase-3 activity, and ROS production in PC12 cells, and protect mesencephalic dopaminergic neurons from MPP^+^-induced toxicity *in vivo* [182] (Figure 3). Tenuigenin (Table 1), the main active component of *Polygala tenuifolia* (Figure 2), improves the survival rate of tyrosine hydroxylase-immunoreactive neurons, reduces dopamine content in the substantia nigra, and abolishes the production of TNF-*α* and IL-1*β* in the lipopolysaccharide- (LPS-) induced PD model [182] (Figure 3). Tenuigenin also protects MMP and significantly increases the expression level of GSH and SOD in 6-OHDA-damaged SH-SY5Y cells [183] (Figure 3). In mechanisms, tenuigenin was demonstrated to inhibit NLRP3 inflammasome activation and intracellular ROS production to increase striatal dopaminergic levels and improve motor impairment in MPTP-induced mice [184] (Figure 3). Onjisaponin B (Table 1) derived from *Radix Polygalae* can induce autophagy and accelerate the removal of neurons with mutant huntingtin and A53T *α*-synuclein via the AMPK-mTOR signaling pathway in PC12 cells [185] (Figure 3). Recently, Peng et al. evaluated the neuroprotective effects of onjisaponin B using MPTP-induced subacute PD mice, and found that it improves motor impairment, attenuates microglia overactivation, and reduces the production of inflammatory factors including TNF-*α*, IL-1*β*, and IL-6 [186] (Figure 3). Through its mechanism, they demonstrated that onjisaponin B inhibits the expression of the p65 subunit of NF-*κ*B complex in the nucleus and attenuates expression of the RhoA and ROCK2 proteins in PD mice [186] (Figure 3).

### 3.14. Polygonum

TSG (2,3,5,4′-tetrahydroxystilbene-2-O-*β*-D-glucoside), an active component of *Polygonum multiflorum* Thunb., has significant antioxidant and free radical-scavenging activities. In multiple cellular PD models, TSG was found to enhance cell viability and inhibit cell apoptosis and ROS production by modulating the JNK, p38, and PI3K-AKT signaling pathway *in vitro* [187–190] (Figure 3). In 6-OHDA-induced PD mice, daily intraperitoneal injection of TSG for 14 consecutive days significantly protects DA neurons from 6-OHDA-induced neurotoxicity and suppresses microglial activation [191]. In MPTP-induced PD mice, TSG ameliorates the injured animal's behavioral ability and dopaminergic neuron loss via restoring the FGF2-AKT and BDNF-TRKB signaling axis in the substantia nigra and corpus striatum [192] (Figure 3). Resveratrol (Table 1) derived from *Polygonum cuspidatum* (Figure 2) also decreases abnormal rotational behavior, the loss and apoptosis of nigral cells, and the levels of total ROS in 6-OHDA-induced PD mice [193] (Figure 3). Juglanin (Table 1), a natural compound extracted from the crude *Polygonum aviculare*, also exhibits anti-inflammatory, antioxidant, and anticancer activities (Figure 3). In 2018, Zhang et al. reported that juglanin treatment also significantly alleviates LPS-caused behavioral and memory impairments and reduces the enhancement of neurodegenerative markers including amyloid-*β* and p-Tau [194]. Through its mechanism, they identified that juglanin reduces LPS-induced production of proinflammatory cytokines via impeding the TLR4/NF-*κ*B pathway [194].

### 3.15. Psoralea

Monoamine oxidase B inhibitors (MAO-BIs) are relevantly used in the early management of PD. The flavanone bavachinin (Table 1) derived from the seeds of *Psoralea corylifolia* L. (Figure 2) ethanol extract effectively reduces MAO-B activity because of its higher affinity, selectivity, and reversibility as an MAO-BIs [195]. Similarly, Zarmouh et al. identified that biochanin-A, a compound from *Psoralea corylifolia* L. seeds, is a potentially reversible and selective MAO-B inhibitor [196]. Isobavachalcone, another component of *Psoralea corylifolia*, effectively remits MPTP-induced PD mice and alleviates neuronal necrosis [197]. In the mechanism, it was reported that isobavachalcone relieves the microglia-mediated inflammation by modulating the NF-*κ*B signaling pathway [197] (Figure 3). Their prenylchalcones isolated from *Psoralea corylifolia* including isobavachalcone, bavachromene, and kanzonol B were also reported to reduce the expression of protein and mRNA of inducible iNOS (nitric oxide synthase) and COX-2 (cyclooxygenase-2) in LPS-activated microglia by blocking the I*κ*B*α* degradation and downregulating NF-*κ*B level [198] (Figure 3).

## 4. India Herbal Medicines and PD

### 4.1. *Withania somnifera*

*Withania somnifera* (WS), also commonly called winter cherry or poison gooseberry, is a medicinal plant belonging to the Solanaceae family. In modern pharmaceutical chemistry research, bioactive molecules including triterpene lactones, alkaloids, tropine, steroidal lactones, and withanolides have been isolated from WS. Of note, withanolides have a similar chemical structure with the ginsenosides derived from *Panax ginseng*, which is why WS is commonly called “Indian ginseng.” Although WS has been used as a medicinal herb in the treatment of many neurological deficits including poor memory, depression, epilepsy, and neurodegeneration in India for more than 5000 years, the strong scientific evidence to support its safe or effective use in treating any disease is still elusive. Therefore, WS is currently not recommended in clinical use at any condition, which is why it is sold as a dietary supplement in many other countries. Previously, the root extract of WS was reported to possess multiple bioactivities such as antiaging, antioxidant, free radical scavenging, anti-tumorous, etc. [199] (Figure 3). In 2014, Prakash et al. investigated the neuroprotective role of MS in the Maneb- (MB-) and paraquat- (PQ-) induced mouse model, also a widely used PD mouse model [200]. Functionally, they found that the ethanol extract of WS roots significantly promotes dopamine secretion in the substantia nigra and the locomotor activity of PD mice. Besides, their findings uncovered that the ethanol extract of WS roots significantly decreased iNOS concentration (oxidative stress) and GFAP protein level (a proinflammatory marker of astrocyte activation) in the brain tissues of PD mice (Figure 3). The extensive oxidative and inflammatory stress in the brain can induce neuron apoptosis, and then gradually cause PD phenotypes. In this sense, MS exhibits PD-alleviated effects in mice in a similar mechanism with most of the neuroprotective Chinese herbal medicines mentioned above.

### 4.2. *Mucuna pruriens*

*Mucuna pruriens* (MP, also named as Lidou in Chinese and lacuna bean in common English) is a tropical leguminous plant that is native to Africa and tropical Asia including southern China and eastern India [80]. All its parts possess valuable medicinal properties. MP produces seed pods containing serotonin and mucunain that frequently causes human skin to itch when touching it, which makes MP notorious. In Indian traditional medicine, MP seeds have been used in the treatment of diseases including aging, rheumatoid arthritis, diabetes, and neurodegenerative diseases, and also as a tonic and aphrodisiac for male virility [201]. The plant of MP naturally contains a relatively high L-DOPA level (~5% of dry weight), making it one of the main sources of L-DOPA [202, 203]. In early 2004, Manyam et al. revealed that MP cotyledon powder treatment significantly restores the endogenous levodopa, dopamine, norepinephrine, and serotonin content in the substantia nigra, which is more efficient than synthetic levodopa treatment [204]. More importantly, a human study by Lieu et al. indicated that the water extract of MP seed powder exhibits less occurrence in the treatment of dyskinesia when compared to standard levodopa treatment [205]. In 2017, Yadav et al. investigated the effect of the ethanol extract of MP on the level of NO in brain tissues and its subsequent contribution to lipid peroxidation in the PQ-induced PD mouse model [206] (Figure 3). Their findings uncovered that the MP ethanol extract protects the dopaminergic neurons in the substantia nigra of PQ-induced PD mice by attenuating iNOS expression, nitrite content, and lipid peroxidation level in injured tissues. Meanwhile, a study by Rai et al. is aimed at investigating the effects of the aqueous extract of MP (100 mg/kg body weight) on neuroinflammation in the brain tissues of MPTP-induced PD mice in a manner of oral administration [207]. In their observation, the MP water extract inhibited NF-*κ*B signaling activity, decreased lipid peroxidation and nitrite level, and promoted pAKT1 activity in MPTP-injured brain tissues, and thereby recovering the behavioral abnormalities of animals. Altogether, MP, regardless of the ethanol or water extract, achieves its neuroprotective effect by the anti-inflammatory and antioxidative activities, which is very similar with almost all Chinese herbal medicines with PD-alleviated activity.

### 4.3. *Tinospora cordifolia*

*Tinospora cordifolia* (TC, commonly called gurjo, heart-leaved moonseed, guduchi, or giloy) belongs to the *Menispermaceae* family that is indigenous to tropical regions of the Indian subcontinent. Throughout the centuries, TC has been widely used as an immunomodulator to cure various infections and antidiabetic drugs in traditional Indian medicine [208]. The ethanol extract of TC has been reported to reduce oxidative stress in injured brain tissues to protect neurons and restore the locomotor activity of 6-OHDA-induced PD rats [209]. Meanwhile, the ethanol extract of TC also improves behavioral ability, alleviates brain injury induced by stress, and decreases inflammatory stress in neurons of sleep-deprivation rats [210]. In 2019, Birla et al. explore the anti-inflammatory activity of the TC aqueous extract on the MPTP-intoxicated PD mouse model [211]. They found that biochemical abnormalities, such as the upregulated TNF-*α* and IL-12/1*β* level of MPTP-intoxicated mice were effectively reversed after the treatment of the TC aqueous extract [211]. Considering that the extensive inflammatory stress can induce dopaminergic neuron apoptosis, the anti-inflammatory activity of the TC aqueous extract naturally endows itself with neuroprotective ability. Therefore, similar to Chinese herbal medicines with PD-alleviated activity, TC also exhibits neuroprotective bioactivity on PD animal models by alleviating oxidative and inflammatory stress in brain tissues.

## 5. Herbal Formulation with Anti-Parkinsonian Activities

Over the past decades, numerous Chinese herbal formulations were investigated in the treatment of PD both on clinical trials and animal experiments, of which some examples are listed in Table 1. *Banxia-Houpo-Tang*, a traditional Chinese medicine, was demonstrated to reduce pneumonia risk in older adults with dementia and alleviate swallowing reflex in PD patients [212, 213]. *Kami-Shoyo-San*, consisting of several medicinal herbs that are known in traditional Chinese medicine, also has effects against tremors of psychotic-induced PD patients [213]. Lu et al. reported that *Bushen-Yanggan-Xifeng-Decoction* improves neuron functions by increasing the striatal DA and 5-HT concentration of PD mice models [214]. *Chuanxiong-Chatiao-Pulvis* significantly improves the motor deficit and attenuates dopaminergic neurodegeneration in MPTP-induced PD mice [215]. In 2008, Jin et al. found that, in MPP^+^-treated PC12 cells, *Huanglian-Jiedu-Decoction* shows protective effects on cells [216]. Studies by independent groups demonstrated that *Liuwei-Dihuang-Pill* protects dopaminergic neurons from MPTP-induced injury in PD mice [217, 218]. Both *in vitro* and *in vivo*, *San-Huang-Xie-Xin-Tang* markedly increases tyrosine hydroxylase-positive neurons in the SNpc and improves the motor activity of MPTP-induced PD mice [219]. *Tianma-Gouteng-Yin* was reported by independent groups to protect dopaminergic neurons from apoptosis induced by oxidation stress in PD rats [178, 220]. *Zhen-Wu-Tang* was evidenced with the ability to maintain DA concentration and DA transporter mRNA level in MPTP-treated rats [221, 222]. Interestingly, *Zhichan-Soup* was indicated to promote NSC differentiation in PD model rats [223, 224]. *Jia-Jian-Di-Huang-Yin-Zi-Decoction*, a classical prescription of Traditional Chinese medicine, attenuates the loss of DA neurons, reverses dopamine depletion, and improves the expression of GDNF (glial-derived neurotrophic factor) of MPTP-lesioned mice [225]. *Bu-ShenJie-Du-Fang*, a specific Chinese herbal complex, has a long history of treating motor impairments similar to PD. Recently, Lie et al. demonstrated that, in the MPP^+^-induced cell model of PD, *Bu-ShenJie-Du-Fang* enhances cell survival by stimulating autophagy [226]. In 2020, *Hua-Feng-Dan*, a traditional Chinese medicine used for neurological disorders, was also proven to alleviate LPS- and rotenone-induced behavioral ability injury, and effectively reverse dopaminergic neuron loss in PD rats [227].

## 6. Conclusions and Perspectives

In modern pharmacology, various bioactivity components (such as sesamin, eleutheroside B, and astragaloside IV) from herbal medicines have been demonstrated to possess antioxidative, anti-inflammatory, and neuroprotective effects both *in vitro* and *in vivo*, indicating that they may exhibit therapeutic effects on PD. Here, we also summarized the recent advances in herbal medicines treating PD, including the bioactive components of herbs, 32 Chinese herbal medicines (belong to 24 genera, such as *Acanthopanax*, *Alpinia*, and *Astragalus*), 22 Chinese traditional herbal formulations, and 3 Indian herbal medicines. In these studies, different extraction methods for plant organs (including root, stem, fruit, and flower) were used to prepare treatment reagents. It should be noted that different extracts (such as 80% ethanol or water) of an herbal medicine may exhibit diverse bioactivities in the same experimental system. Besides, the variations in the therapeutic effects of a drug on PD models are often attributed to the administration dose, route of drugs, and the sources of the drug. Therefore, the standard clinical trials on PD patients are absolutely indispensable before their final clinical use. On the other hand, the pharmaceutic studies on herbal medicines may also promote the development of disease-modifying drugs for PD. For example, Chen et al. reported that in MPTP-mediated neurotoxicity in mice, the nanoparticles of puerarin, a valuable compound to treat PD, is more effective in improving disease-associated behavioral deficits and depletion of dopamine and its metabolites than puerarin only, indicating that nanoparticles represent a potentially viable approach to enhancing the oral absorption of puerarin in the treatment of PD.

## Figures and Tables

**Table 1:** Formulations with PD-alleviating effect in Chinese herbal medicines.

Formulations	Herbal medicines and their contents	References
*Banxia-Houpo-Tang*	6 g *Pinellia ternate* Breitenbach, 3 g *Poria cocos* (Schw.) Wolf, 3 g *Magnolia obovata* Thunberg, 2 g *Perilla frutescens* Britton ar. Acuta Lubo, and 1 g *Zingiber officcinale* Roscoc	[212, 213]
*Bushen-Yanggan-Recipe*	15 g *Rehmanniae Radix* Praeparata, 15 g *Rehmannia glutinosa* Libosch., 15 g *Uncariae Ramulus* Cum Uncis, 15 g *Paeonia lactiflora* Pall., 9 g *Polygoni Multiflori* Radix Praeparata	Cai et al., 2002
*Bushen-Huoxue-Granule*	20 g *Fructus Corni*, 20 g *Rhizoma Acortatarinowii*, 20 g *Radix Polygonimultiflori*, 15 g *Herba Cistanches*, 10 g *Raix Angelicaesinensis*, 15 g *Radix Salviaemiltiorrhizae*, and *Scolopendra* 2 pieces	Yang et al., 2010; [193]; Li et al., 2012; Wang et al., 2014; [112]
*Bu-ShenJie-Du-Fang*	*Rehmannia glutinosa*, *Cistanche deser* ticola, *Paeonia lactiflora* Pall, *Radix Angelica* Sinensis, *Puer ariae Radix*, *Coptidis Rhizoma*, *Scutellariae Radix*, *Antelope Horn* Powder, and *Glycyrrhiza uralensis* with a weight ratio of 5 : 5 : 4 : 4 : 5 : 4 : 4 : 1 : 2	[226]
*Chuanxiong-Chatiao-Pulvis*	12 g *Ligusticum chuanxiong* Hort., 12 g *Schizonepeta tenuifolia* Briq., 6 g *Angelicae Dahuricae* Radix, 6 g *Notopterygii Rhizoma* Et Radix, 6 g *Glycyrrhizae Radix* Et Rhizoma, 3 g *Asari Radix* Et Rhizoma, 4.5 g *Saposhnikovia divaricata* (Turcz.) Schischk., 12 g *Mentha haplocalyx* Briq., 4.5 g green tea	[215]
*Fangji-Dihuang-Decoction*	*Rehmannia glutinosa*, *Cistanche deserticola*, *Paeonialactiflora Pall*, *Radix Angelica* sinensis, *Puerariae Radix*, *Rhizoma Coptidis*, *Radix Scutellariae*, *Antelope Horn* powder, and *Glycyrrhizae Radixina* with a weight ratio of 5 : 5 : 4 : 4 : 5 : 4 : 4 : 1 : 2	Xiong et al., 2019
*Huanglian-Jiedu-Decoction*	9 g *Coptis chinensis* Franch, 6 g *Scutellaria baicalensis* Georgi, 6 g *Phellodendron amurense* Rupr, and 9 g *Gardenia jasminoides* Ellis	Durairajan et al., 2014
A modified formulation of *Huanglian-Jie-Du-Tang*	*Rhizoma coptidis*, *Radix scutellariae*, *Cortex phellodendri*, and *Fructus gardeniae* with a weight ratio of 3 : 2 : 2 : 3	Durairajan et al., 2017
*Hua-Feng-Dan*	10% cinnabar (96% as HgS) and 10% realgar (90% as As4S4), along with other components, such as Jingjie (*Nepeta cataria*), Tianma (*Gastrodia elata*), Jiangchan (*Bombyx batryticatus*), Tiannanxing (*Arisaema erubescens*), Baifuzi (*Aconitum coreanum*), Cangshu (*Atractylodes japonica*), and Quanxie (*Buthus martensii* Karsch)	[10, 227]
*Jia-Jian-Di-Huang-Yin-Zi-Decoction*	*Rehmannia Glutinosa* Libosch, *Cornus Ofcinalis* Sieb. et Zucc, *Morinda Ofcinalis* How, *Cistanche Deserticola* Y.C. Ma, *Angelica Sinensis* (Oliv.) Diels, *Asparagus Cochinchinensis* Merr., *Paeonia Lactiflora* Pall. with weight ratio of 1 : 0.6 : 1 : 1 : 1 : 1 : 1	[114]
*Kami-Shoyo-San*	3 g *Bupleurum falcatum*, 3 g *Paeonia lactiflora* Pall., 3 g *Atractylodes lancea*, 3 g *Angelica acutiloba*, 3 g *Poria cocos* (Schw.) Wolf, 2 g *Gardenia jasminoides* Ellis, 2 g *Paeonia suffruticosa* Andr., 1.5 g *Glycyrrhiza uralensis* Fisch., 1 g *Zingiber officcinale* Roscoc, and 1 g *Menthae arvensis*	Ishikawa et al., 2000
*Liuwei-Dihuang-Pill*	24 g *Rehmanniae Radi*x Praeparata, 12 g *Corni Fructus* Praeparata, 9 g *Paeonia suffruticosa* Andr., 12 g Di*oscorea opposita* Thunb., 9 g *Poria cocos* (Schw.) Wolf, and 9 g *Alisma orientalis* (Sam.) Juzep.	[217, 218]
*San-Huang-Xie-Xin-Tang*	5 g *Coptis chinensis* Franch, 5 g *Scutellaria baicalensis* Georgi, and 10 g *Rheum officcinale* Baill.	[219]
*Shouwu-Shudi-Yin*	-	Tunje et al., 2016
*Tianma-Gouteng-Yin*	9 g *Gastrodia elata* Bl., 12 g *Uncariae Ramulus* cum Uncis, 18 g *Haliotidis Concha*, 9 g *Gardenia jasminoides* Ellis, 12 g *Cyathula officinalis* Kuan, 9 g *Eucommia ulmoides* Oliv., 9 g *Taxillus chinensis* (DC.), 9 g *Polygoni Multiflori* Caulis, 9 g *Fulingshe*, and 9 g *Leonurus japonicas* Houtt.	[144, 178]
*Yeoldahanso Tang*	*Pueraria lobata* (Willd.) Ohwi, *Angelica tenuissima* Nakai, *Scutellaria baicalensis* Georgi, *Platycodon grandiflorum* (Jacq), *Angelicae Dahurica*, *Cimicifuga heracleifolia* Kom, *Raphanus sativa* L., *Polygala tenuifolia* (Willd.), *Acorus gramineus* Soland., and *Dimocarpus longan* Lour. with a weight ratio of 6 : 4 : 2 : 1 : 2 : 2 : 2 : 4 : 6 : 6 in dry weight	Bae et al., 2011/2015
*Zhen-Wu-Tang*	30 g *Paeonia lactiflora* Pall., 10 g *Atractylodes macrocephala* Koidz, 10 g *Typhonium giganteum* Engl., 10 g *Poria cocos* (Schw.) Wolf, 10 g *Zingiber officcinale* Roscoc	[221, 222]
*Zhichan-Soup*	15 g *Astragalus mongholicus*, 12 g *Salvia miltiorrhiza* Bge., 10 g *Gastrodia elata* Bl., 18 g *Uncaria rhynchophylla* (Miq.) Miq. ex Havil, 15 g *Paeonia lactiflora* Pall., 9 g *Cimicifugae Rhizoma*, 10 g *Anemarrhena asphodeloides* Bge.	[224]
DA 9805 exerts	DA-9805 was prepared by extracting three dried plant materials (*Moutan cortex*, *Angelica Dahurica* root, and *Bupleurum* root in a 1 : 1 : 1 mixture) with 90% ethanol on a stirring plate for 24 h at room temperature and fingerprinted using high-performance liquid chromatography.	Jeong et al., 2018
KSOP1009 (a modified formulation of Suhexiang-Wan essential oil)	*Liquidambaris Storax* (Hamamelidaceae) (411 g), *Myristicae Semen* (Myristi caceae) (1642 g), *Ligustici Rhizoma* (Umbel liferae) (2189 g), *Santali Alba* Lignum (Santa laceae) (2009.08, 821 g), *Piperis Longi* Fructus (Piperaceae) (2737 g), *Eugeniae Fructus* (Myrtaceae) (821 g), *Typhae Pollen* (Typhaceae) (1095 g), and roots of *Salvia miltiorrhiza* Bunge (Lamiaceae) (3284 g)	[226]
*Zishen-Pingchan-Granules*	15g Di huang (*Radix Rehmanniae*), 15 g Gouqizi (*Fructus Ly cii*), 20 g Sangjisheng (*Taxillus sutchuenensis* Danser), 15 g Tianma (Rhizoma Gastrodiae), 10 g Jiangchan (*Bombyx batryticatus*), 15 g Ezhu (*Rhizoma Curcum* ae Phaeo caulis), 20 g Baishao (*Radix Paeoniae* Alba), 15 g Tian nanxing (*Rhizoma Arisaematis* Erubescentis), 3 g Quanxie (Scorpio), and 3 g Wugong (Scolopendra)	[127]

## Data Availability

Not applicable.

## Abbreviations

PD: Parkinson's disease
CHM: Chinese herbal medicines
CNS: Central nervous system
SNpc: Substantia nigra pars compacta
ETC: Electron transport chain
GDNF: Glial cell line-derived neurotrophic factor
MPPP: 1-Methyl-4-phenyl-4-propionoxypiperidine
MPTP: 1-Methyl-4-phenyl-1,2,3,6-tetrahydropyridine
MPP^+^: 1-Methyl-4-phenylpyridinium
CNS: Central nervous system
6-OHDA: 6-Hydroxydopamine
DA: Dopamine
dsDNA: Double-strand DNA
15-HpETE-PE: Hp-arachidonoyl phosphatidylethanolamine
UPS: Ubiquitin-proteasome system
NO: Nitric oxide
ROS: Reactive oxygen species
LPO: Lipid peroxide
TNF: Tumor necrosis factor-alpha
JNK1/2: c-Jun N-terminal kinase 1/2
ERK1/2: Extracellular signal-regulated kinase 1/2
LPS: Lipopolysaccharide
MMP: Mitochondrial membrane potential
NF-*κ*B: Nuclear factor kappa-light-chain enhancer of activated B cells
HO-1: Heme oxygenase-1
NSCs: Neural stem cells
ER: Endoplasmic reticulum
iNOS: Inducible nitric oxide synthase.
